# The Spatial Morphology of Intraluminal Thrombus Influences Type II Endoleak after Endovascular Repair of Abdominal Aortic Aneurysms

**DOI:** 10.1016/j.avsg.2019.05.050

**Published:** 2020-07

**Authors:** Zachary L. Whaley, Ismail Cassimjee, Zdenek Novak, David Rowland, Pierfrancesco Lapolla, Anirudh Chandrashekar, Benjamin J. Pearce, Adam W. Beck, Ashok Handa, Regent Lee, Oxford Abdominal Aortic Aneurysm Study, Tim Peto, John Finney, Oxford Regional Vascular Services, Chris R. Darby, Alison Halliday, Linda J. Hands, Dominique P.J. Howard, Patrick Lintott, Tim R. Magee, Andrew Northeast, Jeremy Perkins, Ediri Sideso, Emma Wilton, Ashok Handa

**Affiliations:** 1 Nuffield Department of Surgical Sciences, University of Oxford, Oxford, UK; 2 Division of Vascular Surgery and Endovascular Therapy, University of Alabama at Birmingham, Birmingham, AL

## Abstract

**Introduction:**

Type 2 endoleaks (T2Es) after endovascular repair (EVAR) of abdominal aortic aneurysm (AAA) can lead to sac expansion or failure of sac regression, and often present as a management dilemma. The intraluminal thrombus (ILT) may influence the likelihood of endoleaks after EVAR and can be characterized using routine preoperative imaging. We examined the relationship between preoperative spatial morphology of ILT and the incidence of postoperative T2E.

**Methods:**

All patients who underwent EVAR at the John Radcliffe Hospital (Oxford, UK) were prospectively entered in a clinical database. Computed tomography angiograms (CTAs) were performed as part of routine clinical care. The ILT morphology of each patient was determined using the preoperative CTA. Arterial phase cross-sectional images of the AAA were analyzed according to the presence and morphology of the thrombus in each quadrant. The overall ILT morphology was defined by measurements obtained over a 4-cm segment of the AAA. The diagnosis of T2Es during EVAR surveillance was confirmed by CTAs. The relation between the ILT morphology and T2E was assessed using logistic regression.

**Results:**

Between September 2009 and July 2016, 271 patients underwent EVAR for infrarenal AAAs (male: 241, age = 79 ± 7). The ILT was present in 265 (98%) of AAAs. Mean follow-up was 1.9 ± 1.6 years. The T2E was observed in 77 cases. Sixty-one percent of T2Es were observed within the first week after surgery. The T2E was observed in 50% (3/6) of cases without the ILT (no-ILT). Compared with no-ILT, the presence of circumferential or posterolateral ILTs was protective from T2Es (odds ratio = 0.33 and 0.37; *P* = 0.002 and *P* = 0.047, respectively).

**Conclusions:**

The spatial ILT morphology on routine preoperative CTA imaging can be a biomarker for post-EVAR T2Es. ILTs that cover the posterolateral aspects of the lumen, or circumferential ILTs, are protective of T2Es. This information can be useful in the preoperative planning of EVARs.

## Introduction

The rate of endovascular repair (EVAR) as the choice of treatment for abdominal aortic aneurysms (AAAs) continues to rise, as EVAR possesses many benefits compared to open surgical repair (OSR), including shorter postoperative hospital stays and decreased short-term complications.^1^, ^2^, ^3^ However, the increased long-term mortality and complication risk of EVAR have prevented it from being distinguished as a universally superior treatment to OSR, as aneurysm related and all-cause mortality rates of EVAR converge and eventually surpass those of OSR between 3–8 years post-EVAR.^1^, ^2^, ^3^, ^4^

One of the major complications associated with EVAR is endoleak. Endoleak can cause sac expansion and non-sac regression, in which the aneurysm continues to grow, or does not reduce in size, respectively.^5^, ^6^, ^7^, ^8^, ^9^ Emerging evidence support the notion that ongoing sac expansion after EVAR is independently associated with late mortality.^10^

The intraluminal thrombus (ILT) within AAAs has previously been held as a bystander to the disease.^11^ However, emerging evidence indicates ILT to be biologically active and contains inflammatory cells within a network of canaliculi.^12^, ^13^, ^14^ It is possible that native ILTs within the AAA sac are directly involved in the post-EVAR aneurysm sac remodeling process. This is supported by a magnetic resonance imaging study of patients with nonsac shrinkage after EVAR demonstrated significantly more nonorganized thrombus in patients with an endoleak.^15^ There are prior observations of lower endoleak and sac enlargement rates with more sac surface area coverage by ILT,^16^, ^17^, ^18^ but there is little literature regarding the link between the spatial locations of ILT preoperatively, and the occurrence of endoleaks after EVAR.

In this study, we sought to examine the prevalence, morphology, and anatomical variations of native ILT on the preoperative CT scan, and their relationship to type 2 endoleaks (T2Es). We hypothesized that the spatial morphology of ILT is a determinant of post-EVAR T2Es.

## Methods

The study was conducted as part of the ongoing Oxford Abdominal Aortic Aneurysm study (Ethics approval Ref: SC/0250/13). The study complies with the principles outlined in the Declaration of Helsinki. Each patient gave consent for the use clinical and imaging data for research analyses. We utilized the clinical database (Oxnet Janus), which prospectively registered every patient who underwent EVAR of AAAs at the John Radcliffe Hospital, Oxford, UK.

We included patients who underwent EVAR for elective infrarenal AAAs between September 2009 and July 2016. Patients were only included if they had infrarenal EVAR as treatment for AAA, one available preoperative computed tomography angiogram (CTA) of the aorta and at least one postoperative follow-up scan.

Post-EVAR surveillance includes the use of ultrasound (US) scan and CTAs. When a post-EVAR endoleak is suspected on duplex US scan, a diagnosis is made using CTAs, taking into consideration the arterial and venous phase images. The diagnosis in each case was independently confirmed by vascular radiologists as a part of their reporting of the scans. Every postoperative CTA for each patient was included in this database, and a patient was considered positive for endoleak if any of these reports noted a T2E throughout their follow-up period. Demographic data were gathered using ICD-10 (International Statistical Classification of Diseases and Related Health Problems - 10th revision) codes registered with the patient's electronic medical record.

### Characterization of Intraluminal Thrombus

ILTs were assessed using the preoperative CTA routinely performed as part of the clinical management for preoperative planning. As the AAAs vary in size/length, we sought to objectively measure across a segment of the AAA which captures sufficient information about the AAA sac, but with minimal chance of confounding by other factors such as tortuosity. The AAAs also had various neck lengths, which also influenced the choice of a reference point for analysis.

**Fig. 1:**
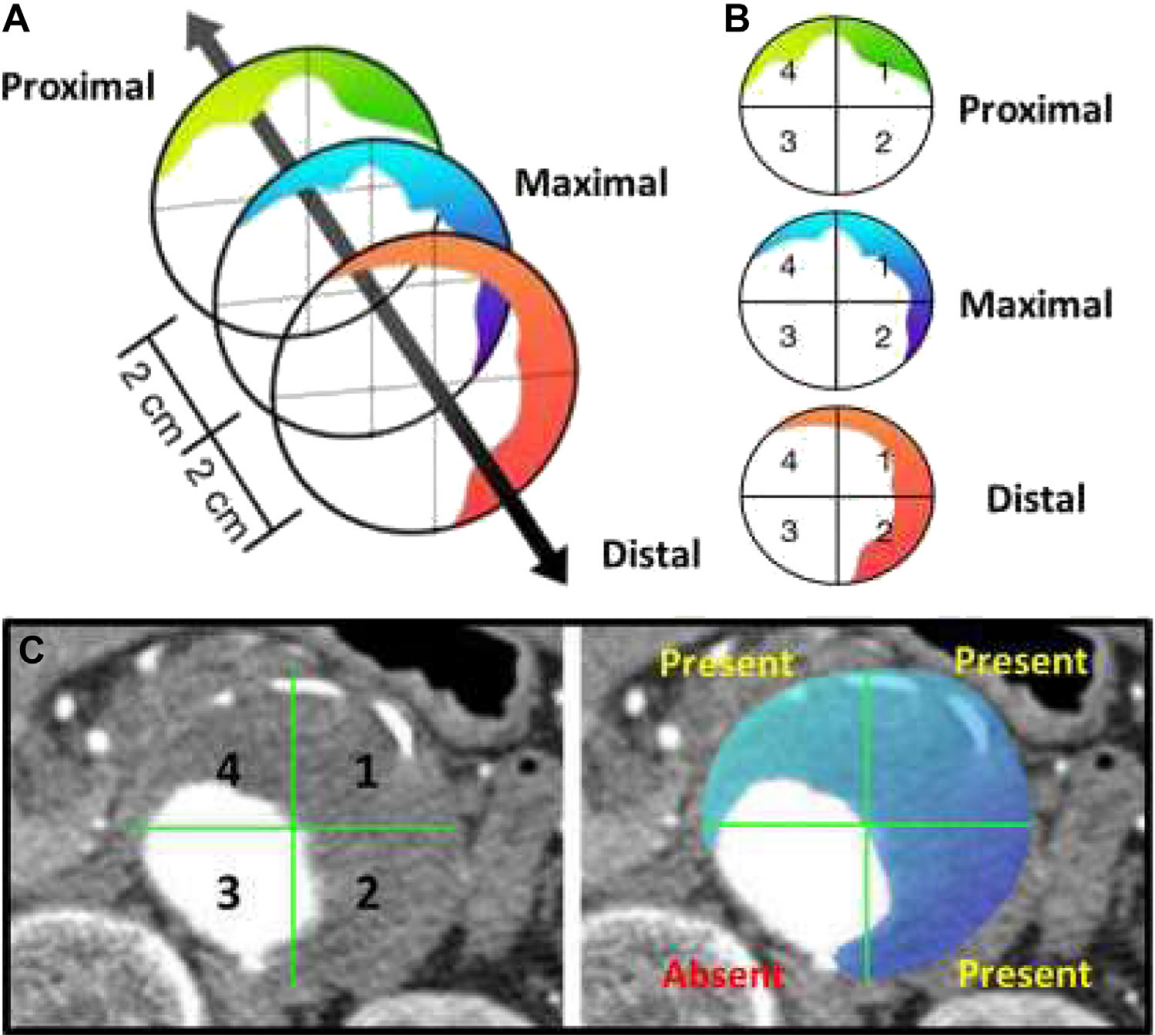
Characterization of intraluminal thrombus. ILTs are assessed using the preoperative CTA routinely performed as part of the clinical management for preoperative planning. The cross-section which contained the maximum anterior-to-posterior (AP) sac size was chosen as the reference section for each patient (“maximal”). In each patient, we included the segments 2 cm proximal and distal to the reference segment (“proximal” and “distal”) to examine the volumetric distribution of ILT within the aneurysm **(A)**. Each axial slice/cross-section within this 4 cm segment was analyzed. For each axial slice, perpendicular AP and transverse lines divide it into 4 quadrants, labeled 1–4 in a clockwise fashion **(B)**. ILT was classified positive for a quadrant if there was visible presence of ILT occupying at least one-third of the quadrant circumference **(C)**.

To standardize the analysis, the cross-section which contained the maximum anterior-to-posterior (AP) sac size was chosen as the reference section for each patient (“maximal”). In each patient, we included the segments 2 cm proximal and distal to the reference segment (“proximal” and “distal”) to examine the spatial distribution of ILT within the aneurysm (Fig. 1A).

The axial slices/cross sections at these 4 cm segment was analyzed. For each axial slice, perpendicular AP and transverse lines divide it into 4 quadrants, labeled 1–4 in a clockwise fashion (Fig. 1B). The ILT was classified positive for a quadrant if there was visible presence of ILT occupying at least one-third of the quadrant circumference (Fig. 1C). The choice to use one-third as a threshold for quadrant positivity was intended to account for a minimal coverage area within the quadrant, while still being large enough to be easily appraised.

**Fig. 2:**
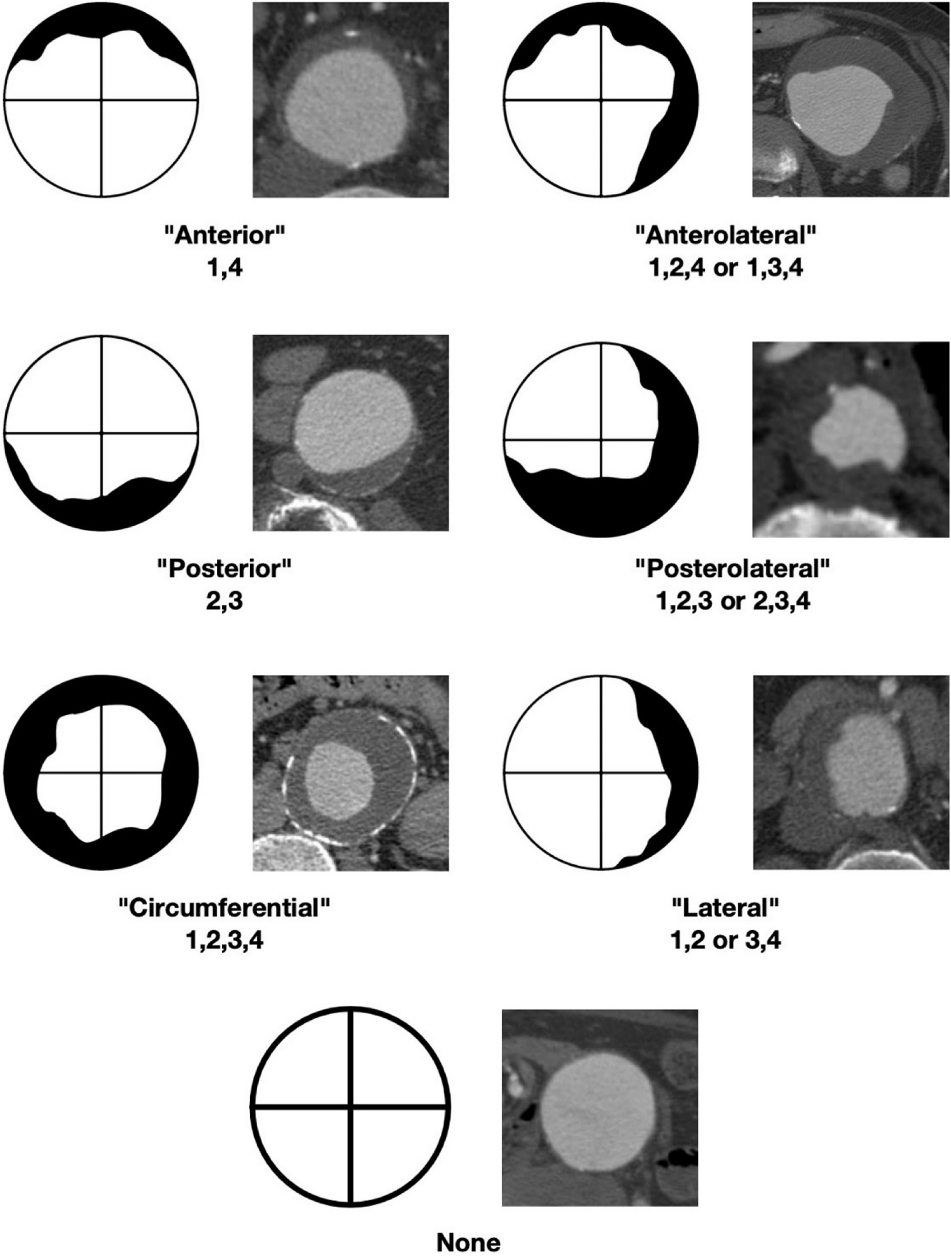
Classification of intraluminal thrombus (ILT). The ILT was considered “anterior,” “posterior,” or “lateral” when the whole AAA ILT was only positive for two adjacent quadrants, based on the location of those quadrants. ILT found in three quadrants were labeled either “anterolateral” or “posterolateral” based on their dominant coverage area. If all four quadrants were positive, the ILT was labeled “circumferential.” AAAs that were positive for ILT, but did not have more than two contiguous positive quadrants in each of the three slices, were considered “amorphous.”

The different ILT types were named based on their coverage of the section, as illustrated by Figure 2. The ILT was considered “anterior,” “posterior,” or “lateral” when the whole AAA ILT was only positive for two adjacent quadrants, based on the location of those quadrants. ILT found in three quadrants were labeled either “anterolateral” or “posterolateral” based on their dominant coverage area. If all four quadrants were positive, the ILT was labeled “circumferential.” AAAs that were positive for ILT but did not have more than two contiguous positive quadrants in each of the three slices were considered “amorphous.”

### Statistical Analysis

Data are described as absolute numbers and percent prevalence (%). Continuous variables are presented as mean ± SD or median with interquartile range. The chi-square test (or Fishers' exact test where applicable) was used to compare frequencies and independence of samples. Correlations were examined by Pearson's or Spearman's rank correlations where appropriate. Multivariate logistic regression using backward selection analyses were used to analyze risk factors influencing outcomes and included all risk factors/demographics. A *P*-value of less that 0.05 was considered statistically significant. All data were analyzed using SPSS v.24 package (IBM Inc.)

## Results

### Demographics of the Cohort

Two hundred seventy-one patients underwent EVAR for infrarenal AAAs (male: 241, average age = 79 ± 7). The ILT was present in 265 (98%) of AAAs. Median follow-up was 1.6 ± 1.6 years. The baseline demographic data are summarized in Table I.

**Table I:** Demographic details of the study cohort

	T2E	No T2E	*P* Value
Number of cases	77	194	
Male (%)	70 (91)	171 (88)	0.51
Diabetes (%)	8 (10)	25 (13)	0.57
Hypertension (%)	52 (68)	121 (62)	0.43
Hypercholesterolemia (%)	18 (23)	41 (21)	0.69
Peripheral vascular disease (%)	15 (19)	42 (22)	0.69
Coronary heart disease (%)	30 (39)	73 (38)	0.84
Stable angina (%)	4 (5)	12 (6)	0.76
MI/ACS (%)	11 (14)	27 (14)	0.94
PCI (%)	10 (13)	16 (8)	0.23
CABG (%)	10 (13)	17 (9)	0.30
Cardiac arrhythmia (%)	15 (19)	37 (19)	0.94
Respiratory disease (%)	23 (30)	50 (26)	0.49
Hepatobiliary disease (%)	6 (8)	7 (4)	0.15
Gastrointestinal disease (%)	17 (22)	49 (25)	0.58
Renal/urinary disease (%)	19 (25)	39 (20)	0.41
Endocrine disease (%)	4 (5)	19 (10)	0.22
Hematological disease (%)	4 (5)	7 (4)	0.55
Musculoskeletal disease (%)	16 (21)	28 (14)	0.20
Neoplasia disease (%)	9 (12)	36 (19)	0.17

MI, myocardial infarction; ACS, acute coronary syndrome; PCI, percutaneous coronary intervention; CABG, coronary arterial bypass graft.

### Prevalence of Type 2 Endoleak

T2E developed in 77 (28%). Of these cases, 47 (61%) were discovered within one week of EVAR. The latest initial diagnosis of T2E in this cohort was documented on day 368 after EVAR. The type of stent graft was recorded in 234 patients in this cohort: Anaconda (Vascutek, *n* = 3); Aorfix (Lombard Medical, *n* = 30); Endurant (Medtronic, *n* = 26); INCRAFT (Cordis, *n* = 3); Nellix (Endologix, *n* = 11); Treovance (Bolton Medical, *n* = 38); Zenith (Cook Medical, *n* = 123). We examined the relation between types of stent graft and T2E. There was no difference between stent-graft type and subsequent development of T2E (chi-square test, *P* = 0.09).

### ILT Morphology

The ILT was present in 265 (98%) patients. These were classified according to the method described. Among these, 20 (8%) were anterior, 65 (24%) were anterolateral, 7 (3%) were posterior, 29 (11%) were posterolateral, 83 (31%) were circumferential, 15 (6%) were lateral, and 46 (17%) were amorphous.

### ILT Morphology and the Association with Type 2 Endoleak

**Fig. 3:**
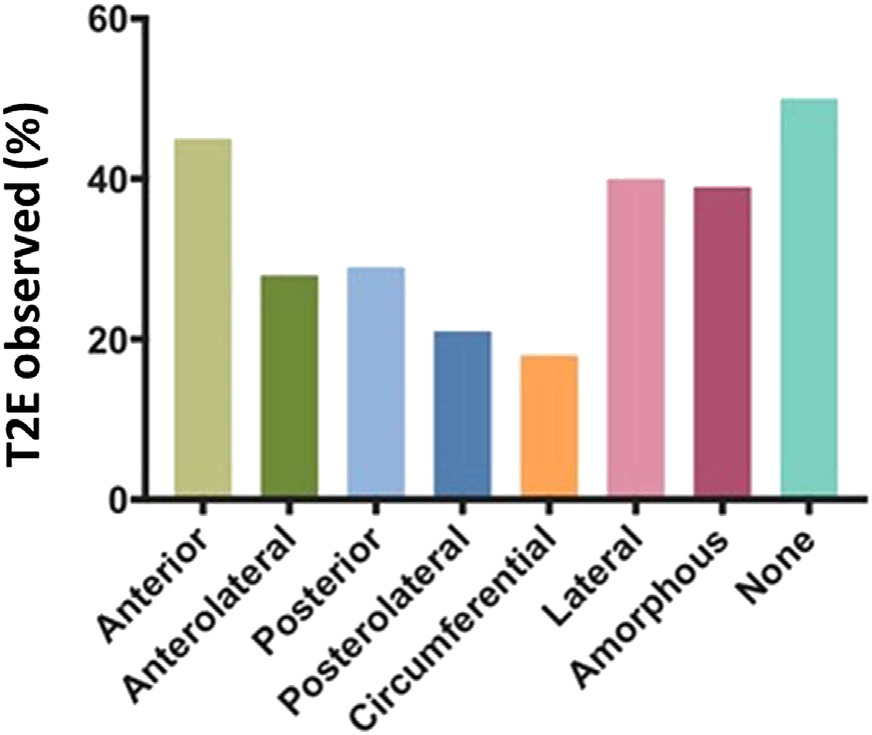
Intraluminal thrombus (ILT) morphology and the association with type 2 endoleak. The T2E was observed in 9/20 (45%) of anterior ILT, 18/65 (28%) of anterolateral ILT, 2/7 (29%) of posterior ILT, 6/29 (21%) of posterolateral ILT, 6/15 (40%) of lateral ILT, 15/83 (18%) of circumferential ILT, 18/46 (39%) of amorphous ILT, and 3/6 (50%) of AAA with no ILT.

The T2E was observed in 9/20 (45%) of anterior ILT, 18/65 (28%) of anterolateral ILT, 2/7 (29%) of posterior ILT, 6/29 (21%) of posterolateral ILT, 6/15 (40%) of lateral ILT, 15/83 (18%) of circumferential ILT, 18/46 (39%) of amorphous ILT, and 3/6 (50%) of AAA with no ILT (Fig. 3). Using a logistic regression to compare different leak rates of ILT type versus no-ILT, a protective benefit was seen in circumferential (OR: 0.33, *P* = 0.002) and posterolateral (OR: 0.37, *P* = 0.047) ILT. When observing individual ILT quadrants, none offers a significant effect on the rate of T2Es. No difference was seen in the ILT between left or right dominant aspects (lateral, anterolateral, and posterolateral types).

## Discussion

With the advancement in endovascular treatment options for AAA, endoleaks as a complication of EVAR are regularly encountered, with T2Es being the most common.^19^ Debate around the insidiousness of T2Es continues, with many considering only AAAs with sac expansion as clinically relevant.^20^ Regardless of this debate, there is consensus on the need for ongoing surveillance in these patients.^20^

The first important observation in this cohort is the higher prevalence of ILTs than previously reported (98% vs. 75%). The traditionally quoted rate originated from a 1982 study by Harter et al. comparing the effectiveness of identifying thrombus in AAA by comparing US versus CT imaging.^21^ This study had 63 participants with various sizes of AAAs, using either US or CT (42 US, 21 CT) as the diagnostic tool. This literature predated the evidence-based guidelines on size threshold for intervention in AAA (e.g. the UK Small Aneurysm Trial^22^ and the ADAM trial^23^), yet it still underpins the common clinical understanding regarding the prevalence of ILT in AAAs. Our cohort represents a contemporary population who underwent endovascular stenting for infrarenal AAAs. Each of the patients had a high-resolution CT scan performed for preoperative planning, which allows for objective assessments for the prevalence of ILT within the AAA sac.

The second important observation from this study is the range of different ILT morphologies present in the AAA sac. Using the cross-sections within a predefined segment of the AAA, centering on the segment with the maximal diameter, we classified the ILT based on anatomical variations (Fig. 2). Although the categories are denoted by the anatomical location of ILT, it also confers biological information. First, the proportional coverage of the AAA lumen by ILT (i.e. its spatial distribution) is reflected by the classification. For example, a posterolateral ILT is likely to occupy higher area than a posterior ILT. Second, the quadrant coverage by an ILT likely corresponds to the underlying aortic side branches that can contribute to the endoleak. For example, one would expect a posterolateral ILT to occupy the wall over the lumbar arteries and perhaps the inferior mesenteric artery, whereas an anterior ILT would not cover the lumbar arteries.

Using this objective classification, we were able to ascertain the association between different ILT types and the event of T2E after EVAR. We observed that posterolateral and circumferential thrombus conferred protection from T2E (22%) compared with the other categories (44%). The lumbar vessels are the common feeding vessels for T2Es. A logical explanation for our finding is that native ILTs occupying the posterolateral aspect of the aneurysm sac prevents retrograde flow from lumbar vessels. This is supported by the observation that those without discernible ILTs within the AAA sac had the highest incidence of T2E after EVAR.

The development of ILT within the aneurysmal sac is incompletely understood. Several theories exist, ranging from flow perturbations within the sac to microdissections of the fragile intima leading to the formation of ILT.^24^ The aorta, unlike the venous system, is a high-flow system and de novo thrombosis in the arterial system is a relatively rare event. Left atrial thrombus in atrial fibrillation and ILT within AAAs are the more common etiologies where high-volume thrombus is in direct contact with the arterial system. Unfortunately, existing aneurysm mouse models do not address the formation of ILT in aneurysms. In all likelihood, the process of ILT formation is multifactorial, and dependent on a combination of local factors, stasis and thrombogeneity within the blood constituents (Virchow's triad).

In patients treated with an EVAR, the potential space between the graft and the thrombus/aneurysm wall is typically filled with new-onset thrombus. The inflow from aortic side branches becomes occluded as the new-onset thrombus evolves and organizes over time. Persistent T2Es are said to occur in patients who have a continuously depressurized sac, either by an open outflow vessel, where the T2E behaves like an arterial venous malformation nidus, or as the sac increases in size. Although this theory is spoken of as fact, it does not have a mechanistic model to test it or supporting literature. Perhaps it is the nature of this new-onset thrombus that determines a persistent T2E, and patients who failed to form ILT in their aneurysm sac preoperatively may then have poor-quality thrombus formation postoperatively, resulting in continuous turnover of their ILT and a T2E.

Several studies aimed to develop models for predicting the risk of developing T2E, using the presence or absence of ILT as a variable.^25^, ^26^, ^27^, ^28^, ^29^, ^30^ None, however, have substratified the ILT into anatomical variations. Our study demonstrates the independent effect ILT morphology has on T2E rates. An advantage exists in regard to location of ILT and total ILT coverage of the AAA sac, with more posterior coverage and circumferential coverage conferring a greater degree of protection from T2Es. Incorporating the spatial definition into these models may improve the accuracy. This remains to be tested.

Increasing the accuracy of preoperative T2E prediction models will allow more nuanced risk stratification. The location-specific coverage area of ILT within AAA's can be assessed in an objective and standardized manner. This would be a useful addition to preoperative risk stratification models for EVAR planning and decision-making on the options for patients with AAA's. Where the anatomy and patient features are suitable for either open repair or EVAR of an AAA, the likelihood of longer term complications, such as persistent T2E and the need for reinterventions, should be comprehensively discussed with the patients.

This study was limited by the absence of preoperative aortic side branch patency within our database, reducing the ability to evaluate the correlation between preoperative versus postoperative patency with respect to ILT type. Due to patients’ choice to pursue follow-up imaging at outside facilities, some patients in this database had limited length of CT follow-up. Although there may have been different techniques of CTAs used during the study period, it is important to note that all the scans are performed as clinical diagnostic scans, performed by accredited radiology departments in the National Health Service (NHS) setting. The results observed therefore reflect the real-life data acquired in the NHS clinical setting and should be robust. The background medical history of individual patients was extracted from the hospital electronic health record system, which did not return the medications for each patient at the time of surgical treatment. Finally, this study only included AAAs treated in the elective setting. Our findings therefore may not apply to EVARs performed for rupture AAAs.

## Conclusion

The spatial morphology of native intraluminal thrombus within the AAA sac influences the onset of T2E after EVAR of AAAs. AAAs that have ILTs that occupy the posterior-lateral aspects or circumferentially within the aneurysm sac have the lowest likelihood for post-EVAR T2Es. This information can be useful in the preoperative planning of EVARs.
